# Supplementary material for: Forward genetic screening using fundus spot scale identifies an essential role for Lipe in murine retinal homeostasis
Source: Commun Biol. 2023 May 17;6:533. doi: 10.1038/s42003-023-04870-7 (PMC10192353; doi:10.1038/s42003-023-04870-7)
Supplement: Supplementary file 2 — Supplementary Information [file 42003_2023_4870_MOESM2_ESM.pdf]

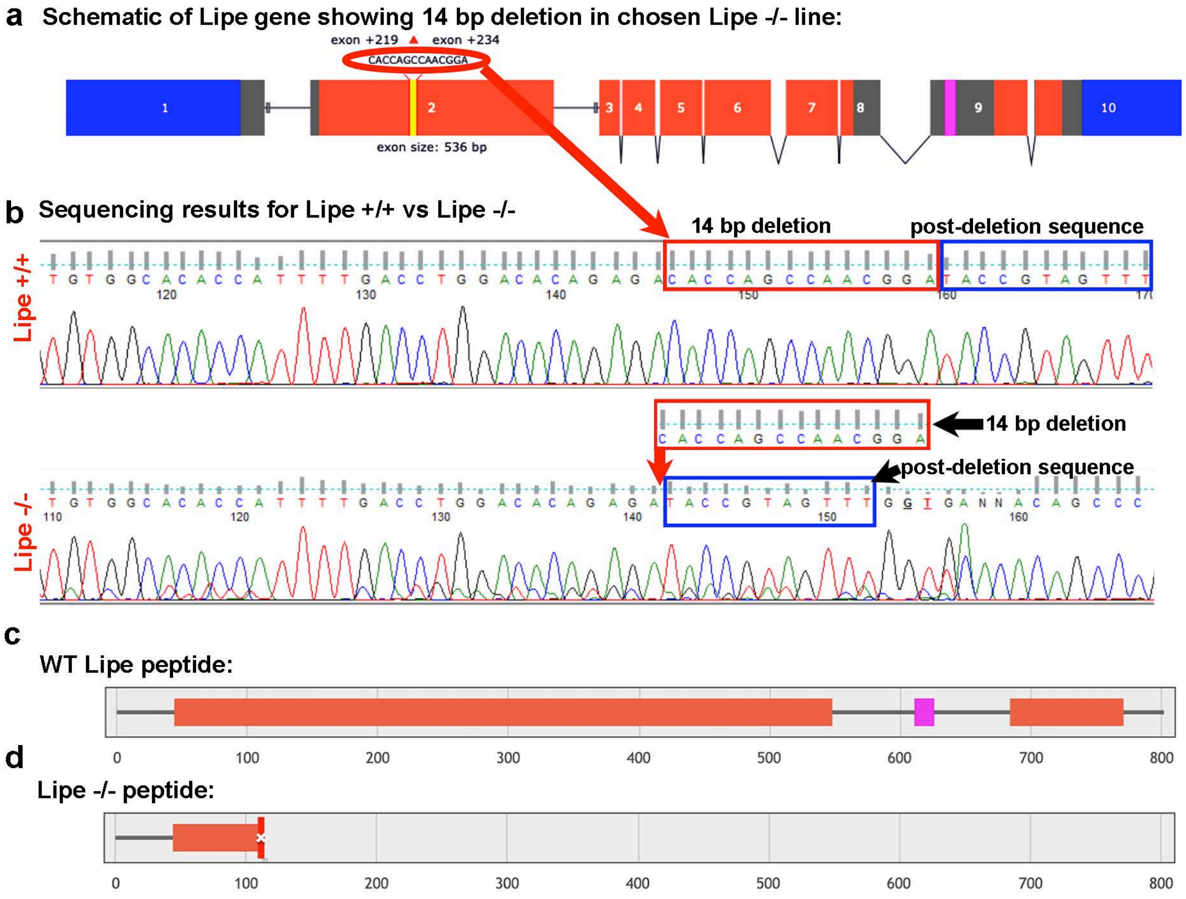

Supplemental Figure S1. Schematic view of Lipe gene showing location of the 14 bp deletion in the CRISPR-generated Lipe-/- mice (a). Examples of Sanger sequencing results for Lipe+/+ and Lipe-/- mice are shown (b), including the 14 bp deletion (red rectangle). Diagrams of the peptide sequence for the wild type Lipe gene (c) and the CRISPR-mutated allele (d) are shown.

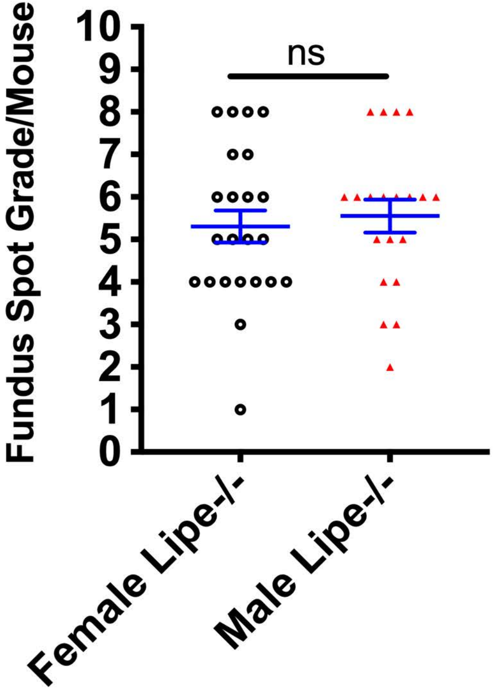

Supplemental Figure S2. No difference was seen between male and female Lipe-/- mice regarding the accumulation of fundus spots. A large cohort of 6-8 m old mice was used for this experiment. Fundus spots grade per mouse (Lipe-/- females, N = 23; Lipe-/- males, N = 20) shown as Means +/- SEM. Two-tailed student's t-test.

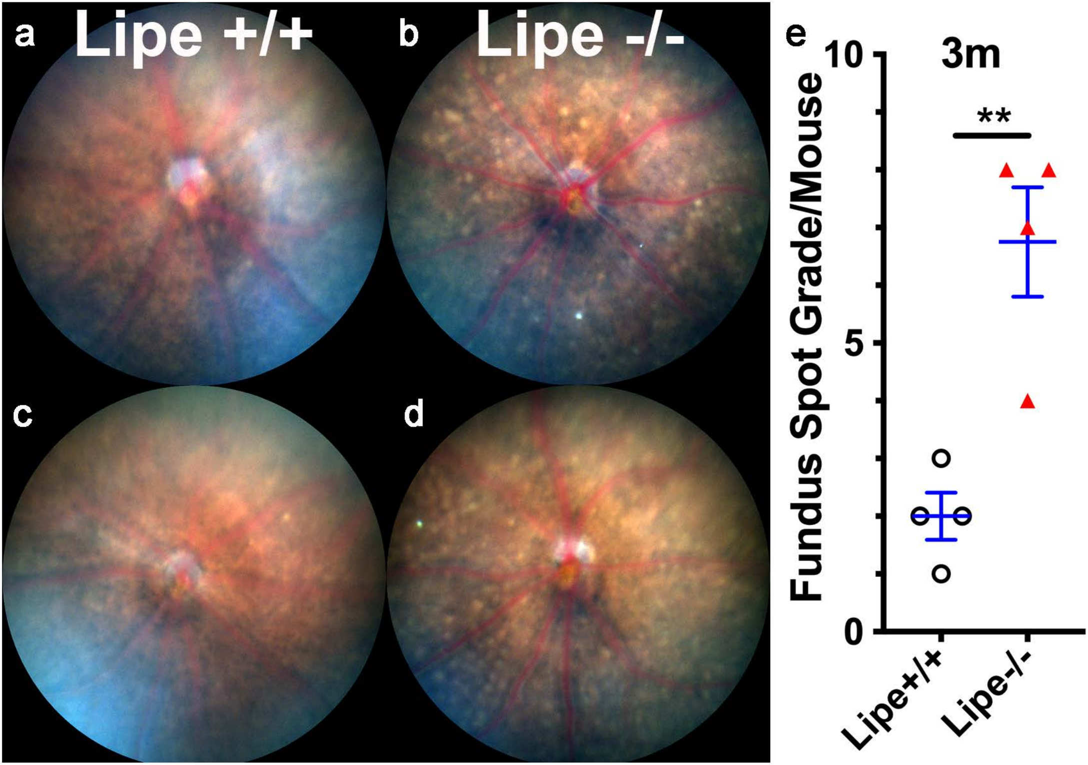

Supplemental Figure S3. A cohort of Lipe-/- CRISPR founder mice was evaluated at 3-4 m of age. By that age we could already observe a statistically significant accumulation of fundus spots. Fundus spots grade per mouse (N = 4 mice per group) shown as Means +/- SEM. Two-tailed student's t-test.

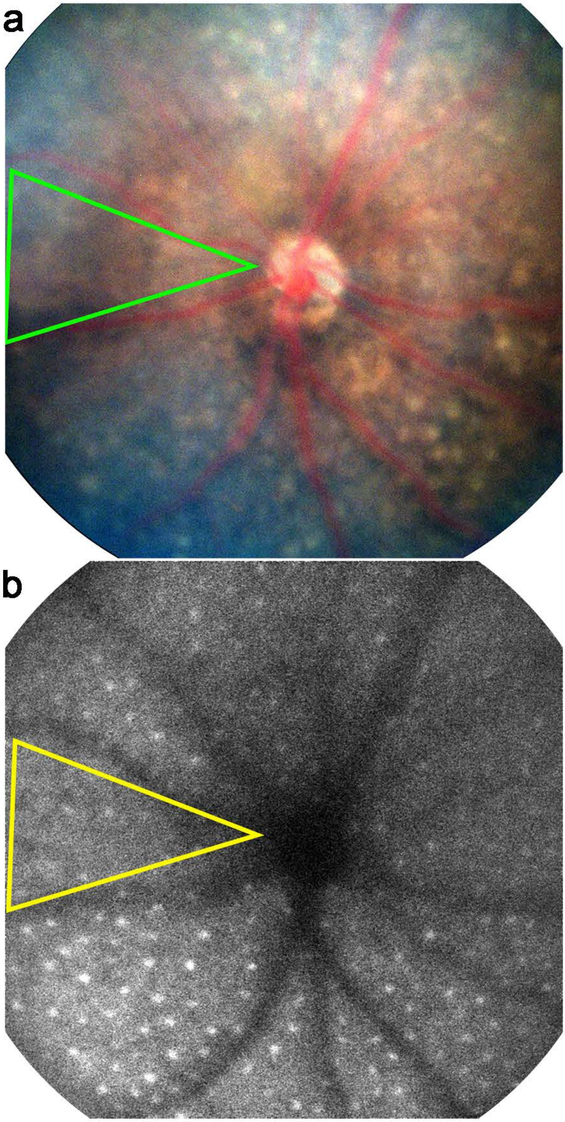

Supplemental Figure S4. Fundus autofluorescence (FAF) imaging in Lipe-/- mice. A representative color fundus photo of a 7 m old Lipe-/- mouse shows a myriad of yellow fundus spots involving all quadrants of the retina (a). FAF was obtained on the same eye and reveals a similar pattern of autofluorescent spots (b). An area with a relatively low number of yellow fundus spots is seen (green triangle in a). Interestingly, this area also shows a paucity of autofluorescent spots in FAF imaging (yellow triangle in b).

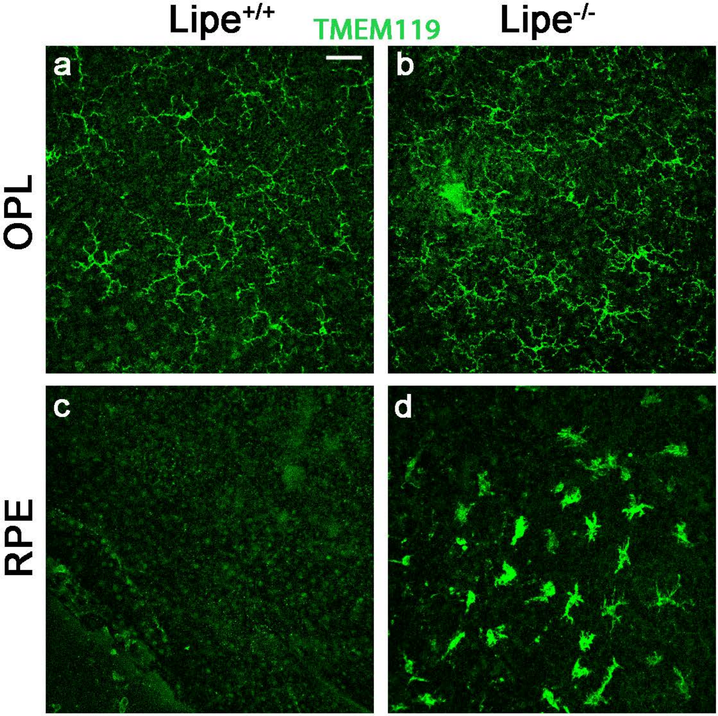

Supplemental Figure S5. Microglial cells in the retina and subretinal space stain positive for TMEM119. Retina (a, b) and RPE (c, d) flat mounts from Lipe+/+ (a, c) and Lipe-/- (b, d) mice were stained using an anti-TMEM119 antibody and imaged using confocal microscopy. For the retina flat mounts, the outer plexiform layer (OPL) was imaged and showed many TMEM119+ cells with small cell bodies and long branching extensions, which were similar in the Lipe+/+ (a) and Lipe-/- (b) mice. However, the RPE flat mounts only showed TMEM119+ cells in the Lipe-/- (d) but not the Lipe+/+ (c) mice. These subretinal microglia had a very different morphology compared to those in the retina, with larger cell bodies and a lower number of extensions which were also shorter. These are typical morphological signs of microglial activation. Scale bar = 50 µm

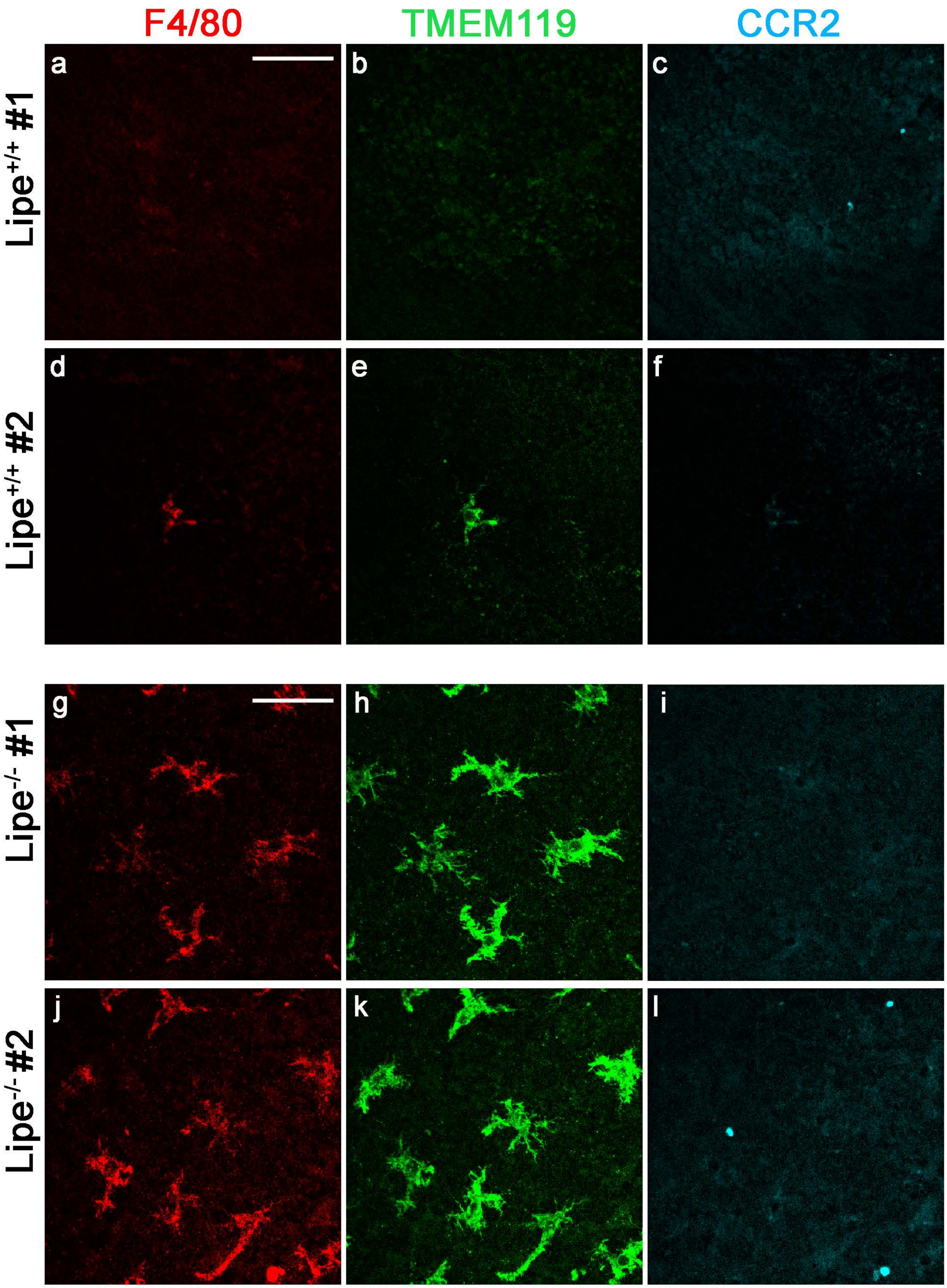

Supplemental Figure S6. The subretinal cells stain with a microglial marker but not a macrophage marker. Additional RPE flat mounts from Lipe+/+ (a-f) and Lipe-/- (g-l) mice were co-stained with F4/80 (a, d, g, j), TMEM119 (b, e, h, k) or CCR2 (c, f, i, l) antibodies. While subretinal cells are only occasionally seen in Lipe+/+ eyes (d-f), they are abundant in Lipe-/- eyes (g-l). These cells stain for the microglial marker TMEM119, but not for the infiltrating macrophage marker (CCR2). Scale bar = 50 µm

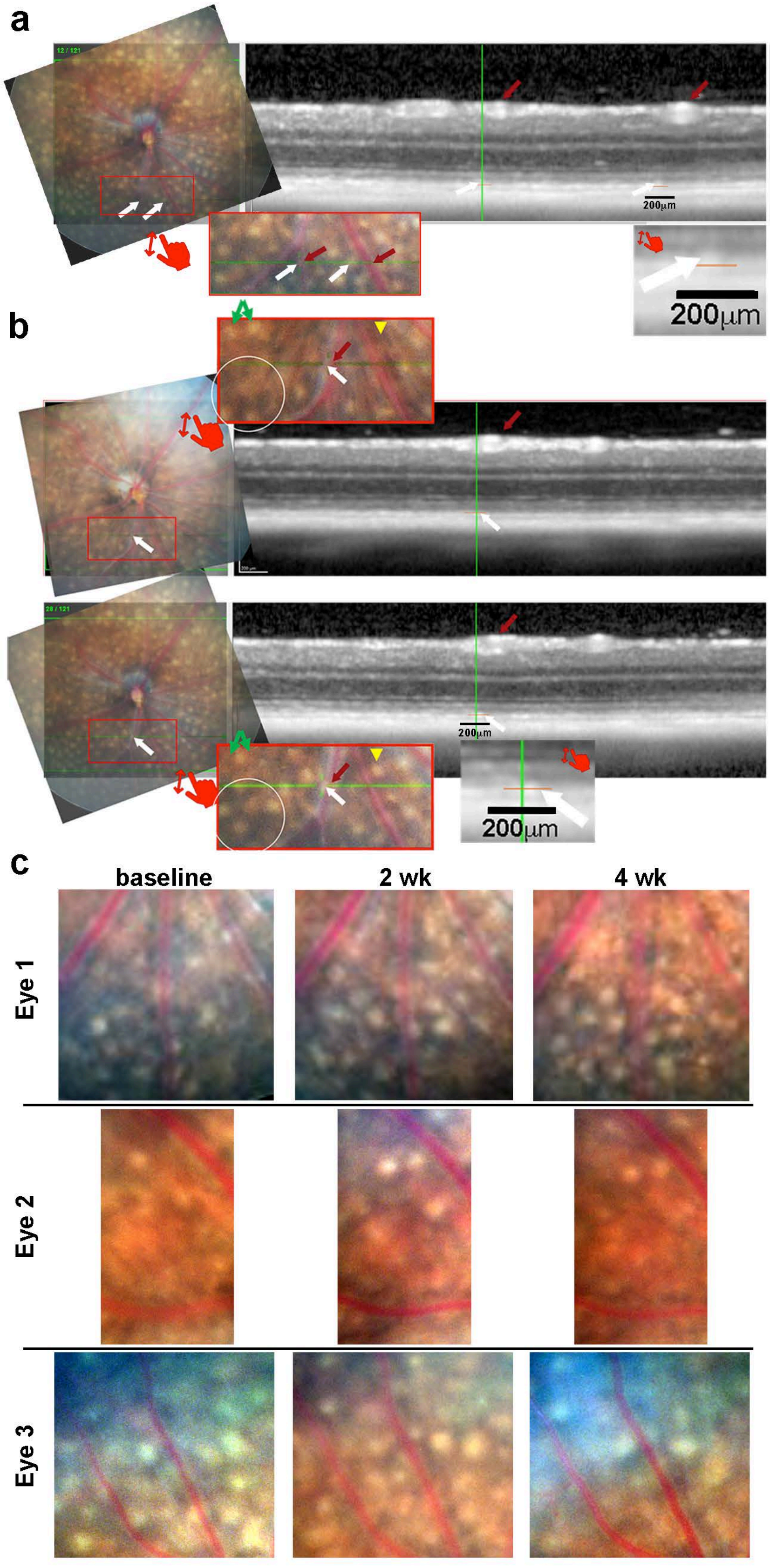

Supplemental Figure S7. The fundus spots correlate to subretinal hyperreflective spots on OCT and are not static. OCT images from Lipe-/- mice were obtained using a Heidelberg Spectralis OCT instrument. a) Using partial transparency mode, color fundus photographs were superimposed on the infrared images that are used to determine the position of the OCT B scans. Evaluation of the OCT images and corresponding fundus images suggested that the yellow spots on color fundus photos (white arrows on color photos) may correspond to small hyperreflective spots just above the RPE/IZ and below the EZ (white arrows on OCT images). Some vessels are labeled with red arrows on the color fundus photo and also in the corresponding location on the OCT images. b) Using the registration feature on the Spectralis OCT we were able to obtain scans of a 7m old Lipe-/- mouse (top half of panel b), and then again in the same location at 10 m of age (bottom half of panel b). Analysis of these images show that at 10 m of age, in the location shown by the white arrow (right next to the vessel labeled with the red arrow) a yellow spot is seen on the color fundus photo and a hyperreflective spot is seen above the RPE in the OCT. These OCT spots were not seen in the images obtained at 7m of age. Furthermore, the small yellow arrowhead shows a new fundus spot, and the green arrows show spots that moved. The white circle shows an area where a significant change in the distribution of spots can be seen. c) Fundus images obtained every 2 wks in 3 different eyes of Lipe-/- mice reveal that the fundus spots change very significantly in a short span of time. At these 2 wk intervals it is common to see new spots appear, some spots disappear, and many spots move to a different location.

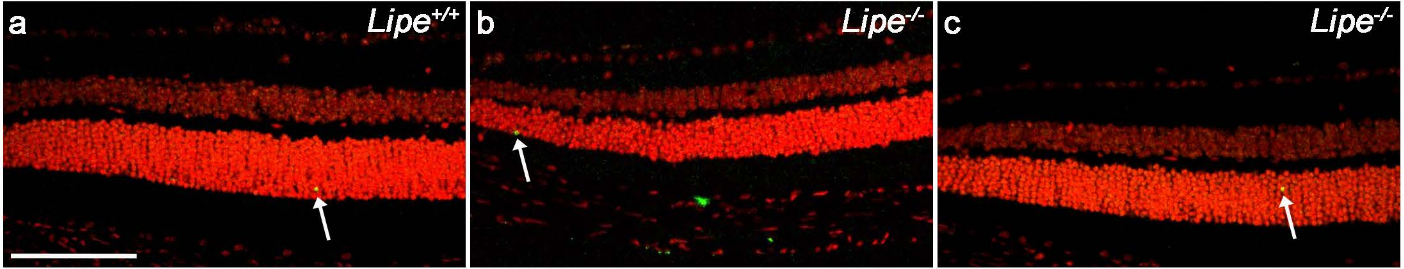

Supplemental Figure S8. No evidence of clumps of apoptotic cells in Lipe-/- mice. TUNEL staining of retinal sections did not reveal any differences between Lipe+/+ and Lipe-/- mice. Both of these mouse lines had only occasional isolated apoptotic cells (white arrows); their number and size would not account for the fundus spots. Scale bar = 100 µm

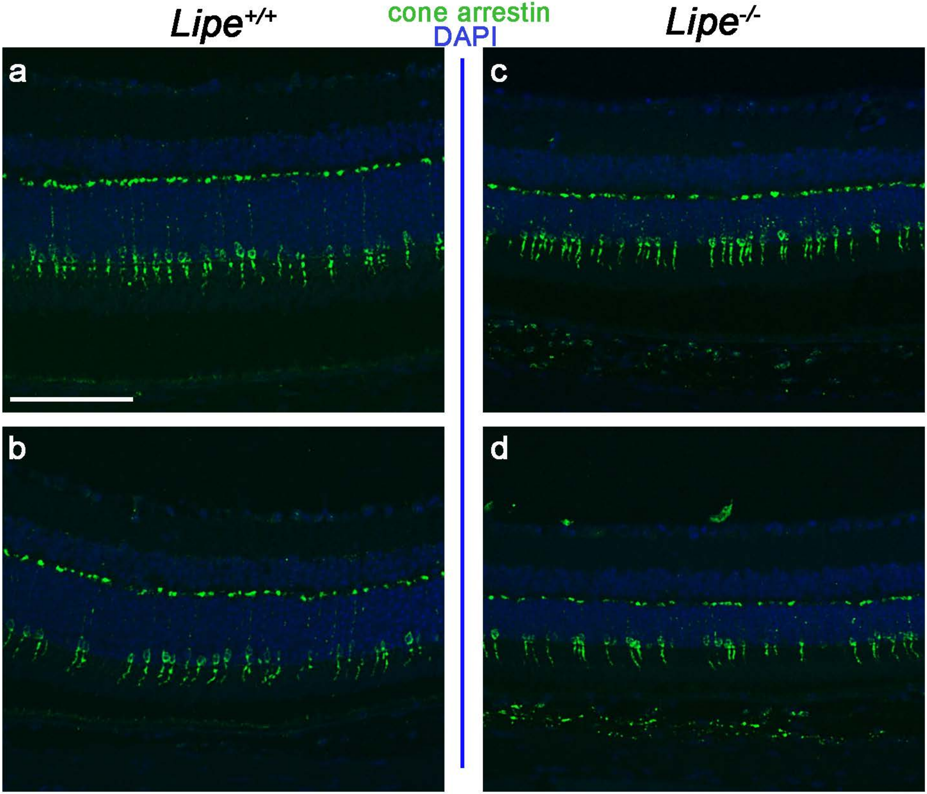

Supplemental Figure S9. No evidence of cone photoreceptor clumping in Lipe-/- mice. Cone arrestin staining of retinal sections did not show any clumping of cone photoreceptors in Lipe-/- mice (c, d) compared to Lipe+/+(a,b) that would explain the fundus spots. Scale bar = 100 µm

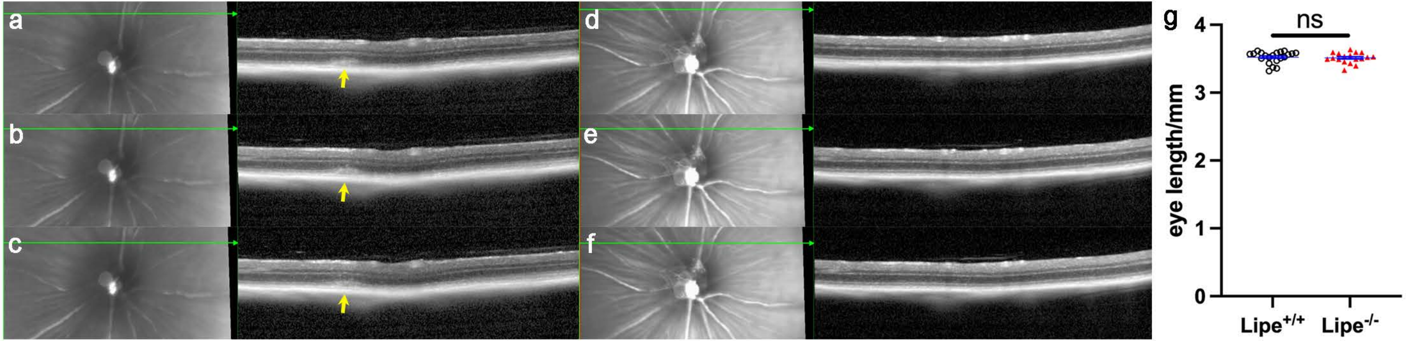

Supplemental Figure S10. A possible choroidal neovascular lesion was detected in a single Lipe-/- mouse. Lipe-/- eyes are of similar size as controls. Three OCT cuts (a-c) obtained with a Heidelberg Spectralis OCT instrument are shown at different levels through a subretinal lesion suggestive of a choroidal neovascular membrane that was seen in a 10 m old Lipe-/- mouse. The exact same levels had been scanned 3 months earlier and did not show the subretinal lesion (d-f). The anteroposterior dimensions of Lipe-/- and Lipe+/+ eyes are similar (g) ruling out myopic degeneration as the source of either the fundus spots or the possible choroidal neovascular membrane. Each symbol represents the average of the anteroposterior measurements of the two eyes of one mouse (n = 18 Lipe-/- and 21 Lipe+/+ mice). Error bars represent Means +/- SEM. Two-tailed student's t-test.

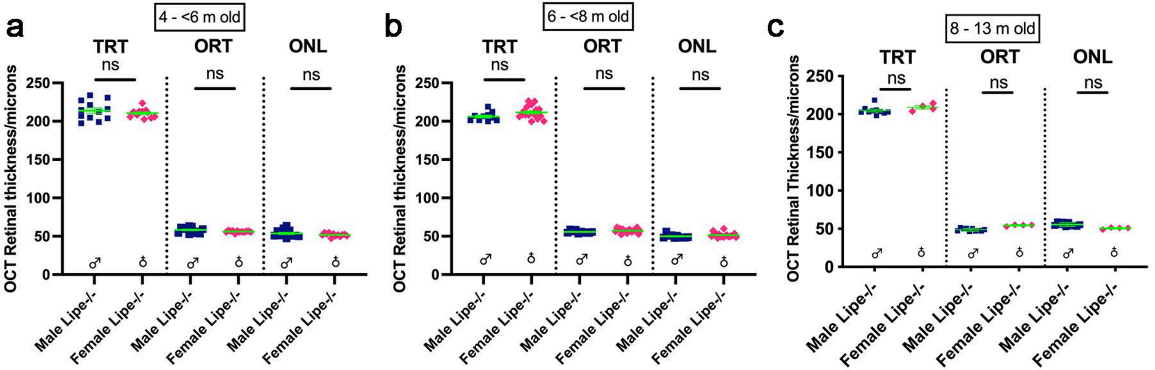

Supplemental Figure S11. No gender difference in OCT parameters were seen in Lipe-/-. Lipe-/- mice were divided into 3 age cohorts (4 - <6 mo, 6 - <8 mo and 8 - 13 mo), and then split by gender. OCT measurements (total retinal thickness or TRT, outer retinal thickness or ORT, and outer nuclear layer or ONL) were obtained for each cohort and males were compared to females (N = 12 OCT images per parameter for each gender). No difference was detected between female and male mice for any of the OCT parameters in any of the age groups. Data are presented as Means +/- SEM and analyzed using a two-tailed student's t-test.

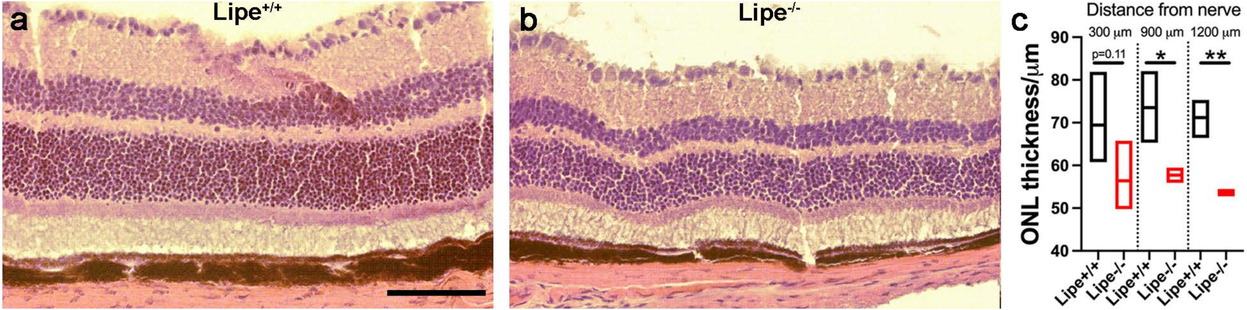

Supplemental Figure S12. Outer nuclear layer thinning was confirmed in cryosections. Eight-month old mice were enucleated (Lipe+/+, N = 4 eyes, Lipe-/-, N = 4 eyes) and the eyes were cryopreserved. Sections were stained with hematoxylin and eosin and imaged at 20x magnification on both sides of the optic nerve head. A masked investigator measured and averaged the ONL thickness at 300, 900 and 1200 microns on either side of the nerve in areas with preserved structural integrity. Data are presented as means and range and analyzed using a two-tailed student's t-test. Scale bar = 100 μm

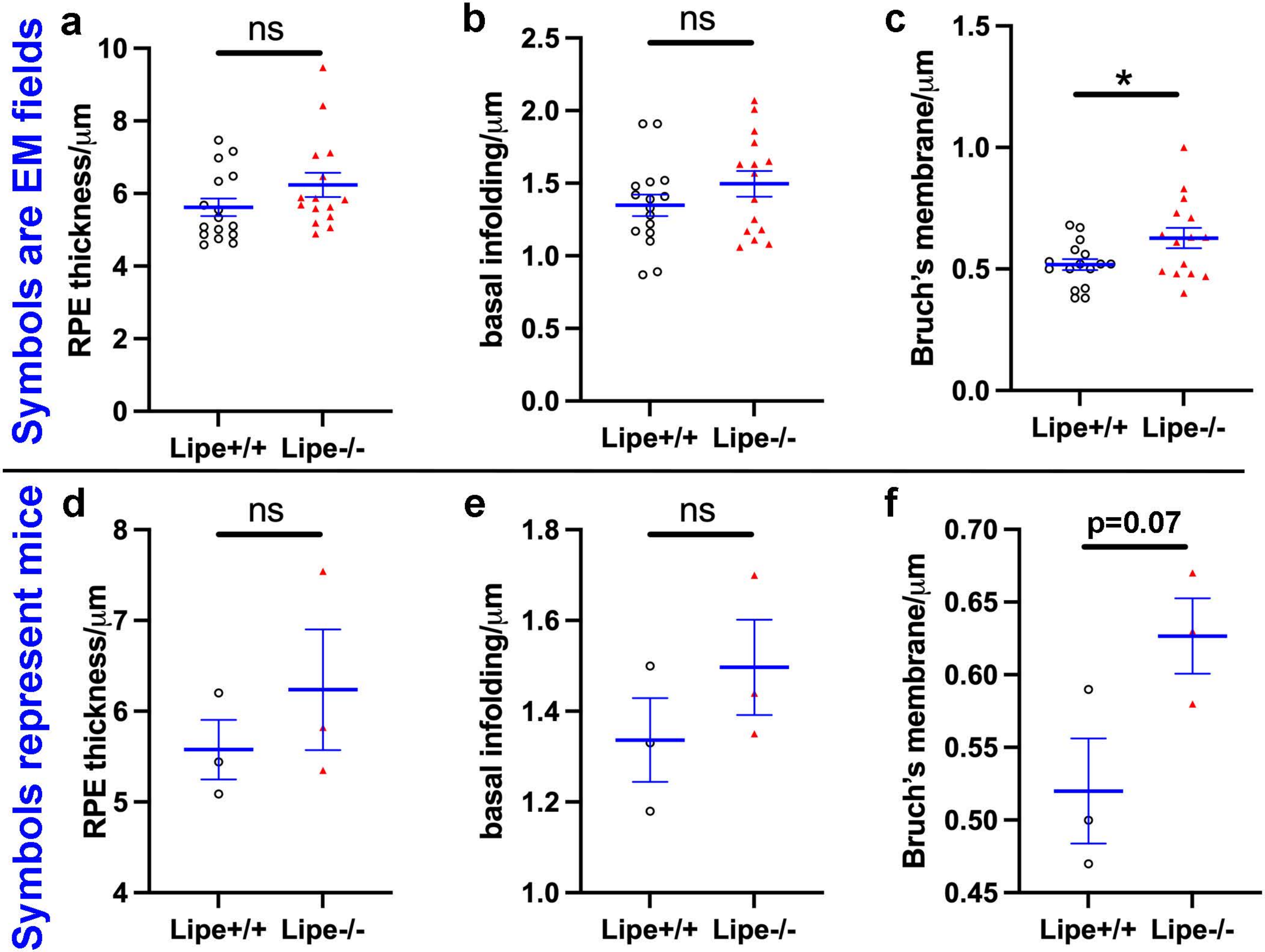

Supplemental Figure S13. Minimal changes in RPE and Bruch's membrane thickness on electron microscopy (EM) imaging of Lipe-/- mice. Electron microscopy images of the RPE and Bruch's membrane of Lipe-/- and Lipe+/+ mice were analyzed to measure RPE thickness (a,d), basal infolding thickness (b,e), and Bruch's membrane thickness (c,f). The analysis was reported per EM field (a-c); n = 20-26 EM fields per mouse line) or per mouse (d-f, n = 3 Lipe-/- and 3 Lipe+/+ mice). While there was no difference in RPE thickness or basal infolding thickness (a,b,d,e), a trend towards increased Bruch's membrane thickness in Lipe-/- mice was seen in the "per-mouse" analysis (f). The difference was significant in the "per-EM field" analysis (c). Data are presented as Means +/- SEM and analyzed using a two-tailed student's t-test. *p<0.05

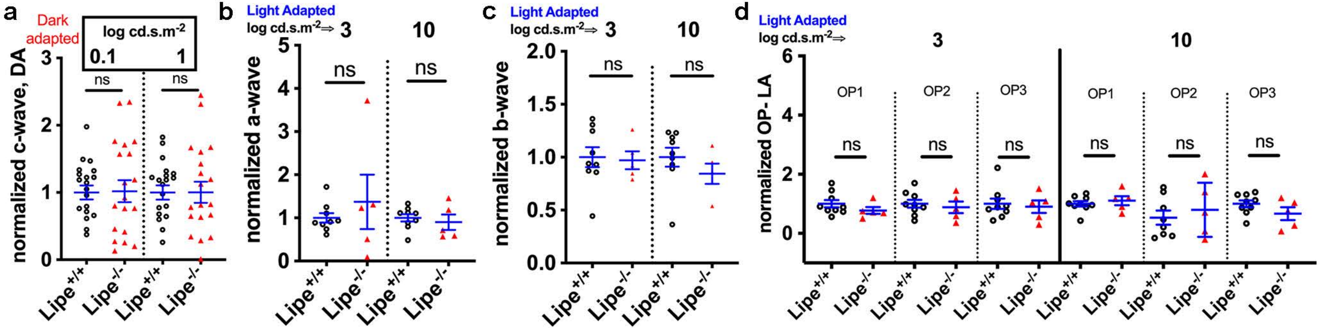

Supplemental Figure S14. Scotopic c-wave and photopic ERG analysis did not show significant differences in Lipe-/- mice. (a) Testing of the scotopic c-wave signal, which may correlate to some aspects of RPE function, was performed in 14 m old Lipe-/- (N = 20) and Lipe+/+ (N = 20) mice. No difference was detected between the two mouse lines. This is consistent with the minimal EM findings in Lipe-/- mice. (b-d) Photopic ERG testing (N = 5 Lipe-/- and 9 Lipe+/+; 1 y old mice) was then performed to test cone responses. The tests included photopic a-wave (b), photopic b-wave (c), and photopic oscillatory potentials (d). No differences were detected in the photopic responses, suggesting that most of the impact to photoreceptors in Lipe-/- mice involves rods rather than cones. Moreover, the preserved oscillatory potentials suggest that the inner retina is functioning well in these mice, which is consistent with the preserved inner retinal thickness on OCT. Data are presented as Means +/- SEM and analyzed using a two-tailed student's t-test.

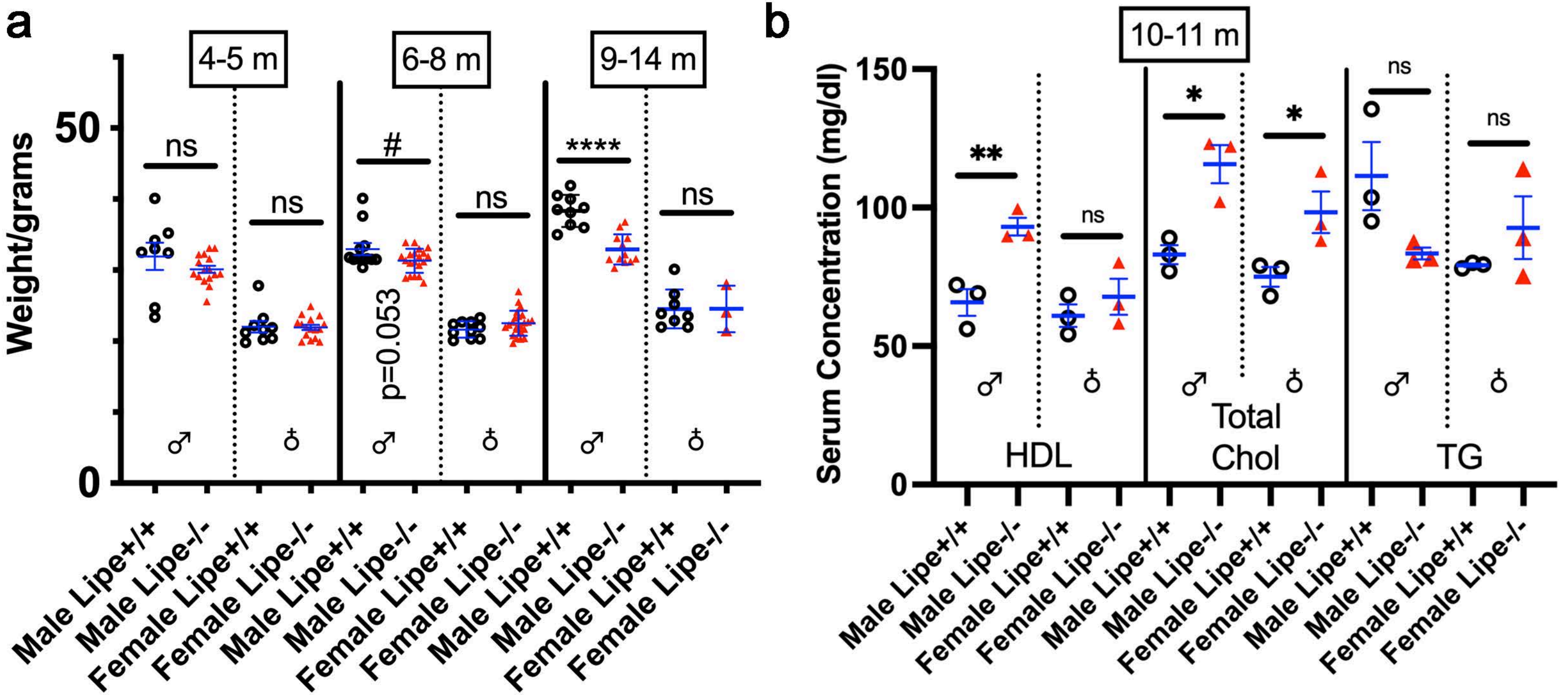

Supplemental Figure S15. Lipe deficiency leads to systemic effects on weight and serum lipid levels. a). Mice were grouped by ages (4-5 mo, 6-8 mo and 9-14 mo) and body weight was measured. Given the gender-related differences in weight, comparisons were made by gender. While no difference in weight was observed between female mouse groups, a statistically significant decrease in weight was observed in the older age group (9-14 mo) of male Lipe-/- mice when compared to Lipe+/+ mice. A trend towards a decrease was also seen in the 6-8m cohort of mice. Body weight was obtained for male (Lipe+/+, N = 8, 12 and 9 mice, Lipe-/-, N = 16, 20 and 12 mice at 4-5 m, 6-8 m, and 9-14 m old respectively) and female (Lipe+/+, N = 9, 10 and 8 mice, Lipe-/-, N = 16, 23 and 3 mice at 4-5 m, 6-8 m, and 9-14 m old respectively). b). In a cohort of 10 mo mice we determined the levels of serum HDL, total cholesterol, and Triglycerides for Lipe+/+ and Lipe-/- mice (N = 3 per genotype per gender). A statistically significant increase in total cholesterol was seen in both male and female Lipe-/- mice when compared to Lipe+/+ mice. HDL cholesterol was also increased in male (but not female) Lipe-/- mice. Data are presented as Means +/- SEM. Two-tailed student's t-test. # $p < 0.1$, *$p < 0.05$, **$p < 0.01$, **** $p < 0.0001$

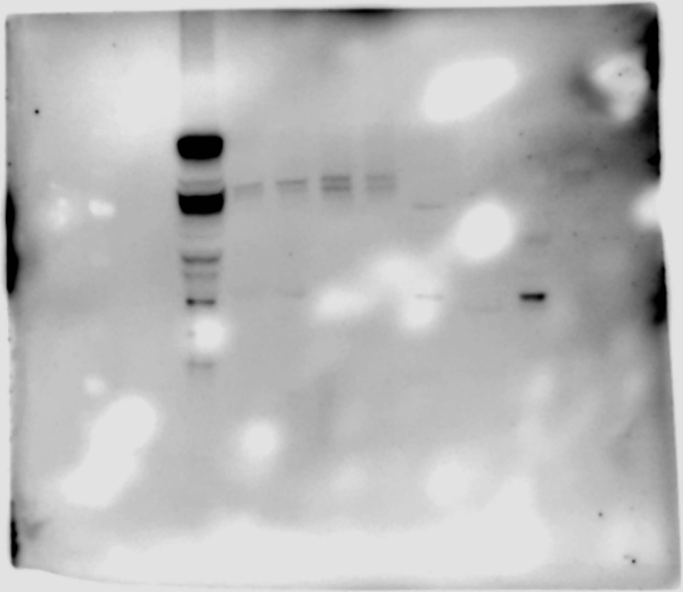

Fig. S16. Unmodified Lipe blot

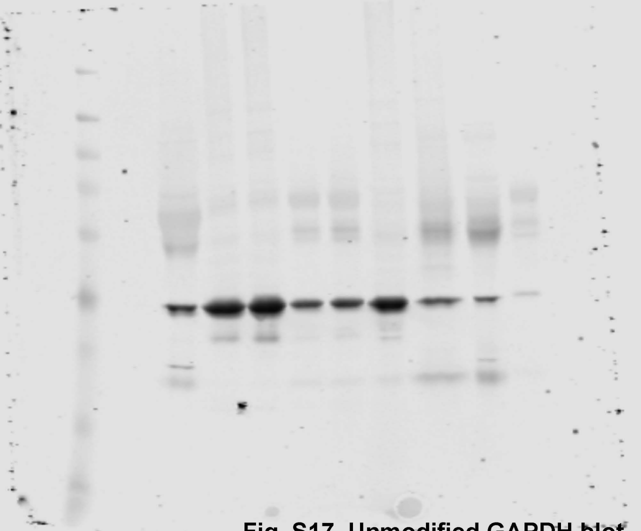

Fig. S17. Unmodified GAPDH blot

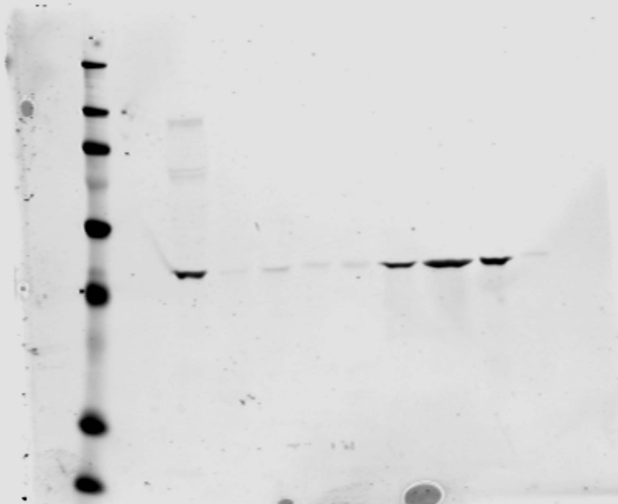

Fig. S18. Unmodified beta actin blot

Table S1. Identified gene-phenotype associations with literature support

#	Gene	Parameter	Homozygous Affected	Mutation Type	Reference
1	Tmem135	1,2,3,4	3/3	NS- PN	[1]
2	Impdh1	1,2,3,4	3/3	NS- PN	[2, 3]
3	Arntl	1,2,3,4	3/3	MS- PrD	[4, 5]
4	Crx	1,2,3,4	9/9	NS- PN	[6-8]
5	Cacna2d4	2,3,4	3/3	CSDS-PN	[9-11]
6	Cngb1	3,4	1/1	MS- PrD	[12, 13]

The fundus spots semiquantitative scale parameter identified 6 genes that have been reported to be associated to retinal development or function. The bottom gene was found based on a single homozygous mouse. The parameters examined were: 1 = BM to ELM thickness on OCT, 2 = BM to ILM thickness on OCT, 3 = ONL thickness on OCT and 4 = Fundus spot scale. Abbreviations: MS - missense, NS - nonsense, CSDS – critical splice donor site, PN – probably null, PrD – probably damaging.

References from Table S1

1. Lee, W.H., et al., *Mouse Tmem135 mutation reveals a mechanism involving mitochondrial dynamics that leads to age-dependent retinal pathologies.* Elife, 2016. **5**.
2. Birtel, J., et al., *Clinical and genetic characteristics of 251 consecutive patients with macular and cone/cone-rod dystrophy.* Sci Rep, 2018. **8**(1): p. 4824.
3. Bowne, S.J., et al., *Why do mutations in the ubiquitously expressed housekeeping gene IMPDH1 cause retina-specific photoreceptor degeneration?* Invest Ophthalmol Vis Sci, 2006. **47**(9): p. 3754-65.
4. Baba, K., et al., *Removal of clock gene Bmal1 from the retina affects retinal development and accelerates cone photoreceptor degeneration during aging.* Proc Natl Acad Sci U S A, 2018. **115**(51): p. 13099-13104.
5. Sawant, O.B., et al., *The Circadian Clock Gene Bmal1 Controls Thyroid Hormone-Mediated Spectral Identity and Cone Photoreceptor Function.* Cell Rep, 2017. **21**(3): p. 692-706.
6. Assawachananont, J., et al., *Cone-rod homeobox CRX controls presynaptic active zone formation in photoreceptors of mammalian retina.* Hum Mol Genet, 2018. **27**(20): p. 3555-3567.
7. Griffith, J.F., M.J. DeBenedictis, and E.I. Traboulsi, *A novel dominant CRX mutation causes adult-onset macular dystrophy.* Ophthalmic Genet, 2018. **39**(1): p. 120-124.
8. Tran, N.M., et al., *Mechanistically distinct mouse models for CRX-associated retinopathy.* PLoS Genet, 2014. **10**(2): p. e1004111.
9. Kerov, V., et al., *alpha2delta-4 Is Required for the Molecular and Structural Organization of Rod and Cone Photoreceptor Synapses.* J Neurosci, 2018. **38**(27): p. 6145-6160.
10. Wycisk, K.A., et al., *Structural and functional abnormalities of retinal ribbon synapses due to Cacna2d4 mutation.* Invest Ophthalmol Vis Sci, 2006. **47**(8): p. 3523-30.
11. Wycisk, K.A., et al., *Mutation in the auxiliary calcium-channel subunit CACNA2D4 causes autosomal recessive cone dystrophy.* Am J Hum Genet, 2006. **79**(5): p. 973-7.
12. Huttl, S., et al., *Impaired channel targeting and retinal degeneration in mice lacking the cyclic nucleotide-gated channel subunit CNGB1.* J Neurosci, 2005. **25**(1): p. 130-8.
13. Winkler, P.A., et al., *A large animal model for CNGB1 autosomal recessive retinitis pigmentosa.* PLoS One, 2013. **8**(8): p. e72229.

Table S2. Materials and Resources

Reagent Type	Name, description, dilution	Source, Reference	Cat. No
Antibody – Primary, IHC	IBA1 Polyclonal, 1:600	Wako Chemicals USA	019-19741
Antibody – Primary, IHC	CD16/CD32 Polyclonal, 1:25	BD pharmaceutical	553142
Antibody – Primary, WB	Lipe/HSL Polyclonal, 1:1000	Cell Signaling Technology	4107S
Antibody – Primary, IHC	Cone Arrestin, 1:250	Sigma-Aldrich	Ab15282
Antibody – Primary, IHC	TMEM119, 1:200	Novus Biologicals, LLC	NBP3-13355
Antibody – Primary, IHC	CCR2, 1:100	Novus Biologicals, LLC	NBP2-35334
Antibody – Primary, IHC	F4/80, 1:100	ThermoFisher Scientific	14-4801-81
TUNEL kit	DeadEnd Flurometric TUNEL system	Promega	G3250
Antibody – Secondary, IHC	Goat anti-Rat IgG (H+L) Alexa Fluor™ 488, 1:200	ThermoFisher Scientific	A11006
Antibody – Secondary, IHC	Goat anti-Rabbit IgG (H+L) Alexa Fluor™ 594, 1:200	ThermoFisher Scientific	A11012
Antibody – Secondary, IHC	Goat anti-rat IgG (H+L) Alexa Fluor™ 568, 1:200	ThermoFisher Scientific	A11077
Antibody – Secondary, IHC	Goat anti-mouse IgG1 Alexa Fluor™ 488, 1:200	ThermoFisher Scientific	A21121
Antibody – Secondary, IHC	Goat anti-rabbit IgG (H+L) Alexa Fluor™ 633, 1:200	ThermoFisher Scientific	A21070
Antibody – Secondary, WB	HRP chemiluminescent secondary antibody, 1:15000	LI-COR Biosciences	926-80011
Antibody – Secondary, WB	Western HRP substrate	ThermoFisher Scientific	34094
Blocking buffer, WB	Blocking buffer	LI-COR Biosciences	927-70001
Camera	Axiocam Mrm	Carl Zeiss AG	
Color CCD camera	Optronics Microfire	Optronics, Goleta, CA, USA	
Diagnostic imaging	Spectralis® OCT	Heidelberg Engineering, Heidelberg, Germany	
ERG software	Diagnosys Espion	Diagnosys, Inc	
Gel	Tris-Glycine gel	ThermoFisher Scientific	XP04205BOX
Gene editing Reagent	CRISPR-Cas9	Integrated DNA Technologies	
Genetic reagent-mice	C57BL/6J (B6J) mice	Jackson Labratories	000664
ISH detection	Chromogenic kit	Advanced Cell Diagnostics (ACD), Hayward, CA USA	322350
Image processing	ImageJ	Fiji system software	
Imaging software	Amersham Imager 600	Amersham Biosciences, Piscataway, NJ	
Lipid standards	Cholesterol, cholesterol esters, triacylglycerols	Avanti Polar Lipids, Inc. and Nu-Check, Inc.	
Liquid chromatography	ultra-high performance reverse phase liquid chromatography	Waters Acquity M-Class UPLC system, Waters Corporation	
Liquid gel	GenTeal liquid gel	Novartis, East Hanover, NJ	
Media	M16 medium	Sigma-Aldrich	M7292
Microscope	Leica DMI 3000 microscope	Leica Microsystems, Inc. Danaher Co. Wetzlar, Germany	
Microscope	Confocal laser scanning microscope, Leica TCS SP8	Leica Microsystems, Inc. Danaher Co. Wetzlar, Germany	
Microscope	Observer D1	Carl Zeiss AG	
Protein concentrators	Protein Concentrator, 3K MWCO, 0.5 ml	ThermoFisher Scientific	88512
Protein assay kit	Pierce™ BCA kit	ThermoFisher Scientific	23225

Retinal imaging	Micron IV and OCT2	Phoenix Micron, Inc. Bend, OR	
RNAscope	Lipe probe<br>Sfxn3 probe<br>DapB probe	Advanced Cell Diagnostics (ACD), Hayward, CA USA	435971<br>846951<br>310043
Scotopic ERG system	Celeris System	Diagnosys LLC, MA, USA	
Sequencing system	Illumina HiSeq 2500	Illumina Innovative Technologies	HiSeq 2500
Software	Linkage Analyzer	Wang et al [22]	
Tissue lysis buffer	T-PER	ThermoFisher Scientific, Rockford, IL, USA)	78510
Transmission EM	JEOL 1200EX II	JEOL USA, Inc., Peabody, MA, USA	
Compound microscope	Leica DM2000	Leica Microsystems, Inc. Danaher Co. Wetzlar, Germany	
OptoMotry System	OptoMotry HD system	Cerebral Mechanics, Inc. Lethbridge, AB, Canada	
Digital caliper	Kynup digital caliper	eVatmaster consulting GmbH, Germany	
