# Supplementary material for: Forward genetic screening using fundus spot scale identifies an essential role for Lipe in murine retinal homeostasis
Source: Commun Biol. 2023 May 17;6:533. doi: 10.1038/s42003-023-04870-7 (PMC10192353; doi:10.1038/s42003-023-04870-7)
Supplement: Supplementary file 3 — Reporting Summary [file 42003_2023_4870_MOESM3_ESM.pdf]

nature portfolio

Corresponding author(s): Rafael L. Ufret-Vincenty, MD

Last updated by author(s): Mar 6, 2023

Reporting Summary

Nature Portfolio wishes to improve the reproducibility of the work that we publish. This form provides structure for consistency and transparency in reporting. For further information on Nature Portfolio policies, see our Editorial Policies and the Editorial Policy Checklist.

Statistics

For all statistical analyses, confirm that the following items are present in the figure legend, table legend, main text, or Methods section.

n/a	Confirmed	
☐	☒	The exact sample size (n) for each experimental group/condition, given as a discrete number and unit of measurement
☐	☒	A statement on whether measurements were taken from distinct samples or whether the same sample was measured repeatedly
☐	☒	The statistical test(s) used AND whether they are one- or two-sided<br>*Only common tests should be described solely by name; describe more complex techniques in the Methods section.*
☐	☒	A description of all covariates tested
☐	☒	A description of any assumptions or corrections, such as tests of normality and adjustment for multiple comparisons
☐	☒	A full description of the statistical parameters including central tendency (e.g. means) or other basic estimates (e.g. regression coefficient) AND variation (e.g. standard deviation) or associated estimates of uncertainty (e.g. confidence intervals)
☒	☐	For null hypothesis testing, the test statistic (e.g. F, t, r) with confidence intervals, effect sizes, degrees of freedom and P value noted<br>*Give P values as exact values whenever suitable.*
☒	☐	For Bayesian analysis, information on the choice of priors and Markov chain Monte Carlo settings
☒	☐	For hierarchical and complex designs, identification of the appropriate level for tests and full reporting of outcomes
☒	☐	Estimates of effect sizes (e.g. Cohen's d, Pearson's r), indicating how they were calculated

Our web collection on statistics for biologists contains articles on many of the points above.

Software and code

Policy information about availability of computer code

Data collection	No custom code or algorithms were generated in this study.<br>MassLynx, Progenesis QI, and EZinfo (all purchased through Waters Corporation) were used for lipid data collection and analysis.<br>Diagnosys, Inc (ERG), Progress software (V.1.1.8.159, In-situ hybridization images)
Data analysis	No custom code or algorithms were generated in this study.<br>MassLynx, Progenesis QI, and EZinfo (all purchased through Waters Corporation)<br>Linkage Analyzer and Candidate Explorer are publicly accessible at https://mutagenetix.utsouthwestern.edu/linksplorer/candidate.cfm.<br>Diagnosys, Inc (ERG), Fiji/ImageJ Software (Fiji: ImageJ, with "Batteries Included") , SigmaStat (v.3.5)<br>Microsoft Excel v. 16.63.1

For manuscripts utilizing custom algorithms or software that are central to the research but not yet described in published literature, software must be made available to editors and reviewers. We strongly encourage code deposition in a community repository (e.g. GitHub). See the Nature Portfolio guidelines for submitting code & software for further information.

Data

Policy information about availability of data

All manuscripts must include a data availability statement. This statement should provide the following information, where applicable:

- Accession codes, unique identifiers, or web links for publicly available datasets
- A description of any restrictions on data availability
- For clinical datasets or third party data, please ensure that the statement adheres to our policy

All data supporting this work are provided within the Article and Supplementary Files, or available from the corresponding authors upon request.

Human research participants

Policy information about studies involving human research participants and Sex and Gender in Research.

Reporting on sex and gender	No human subjects.
Population characteristics	No human subjects.
Recruitment	No human subjects.
Ethics oversight	No human subjects.

Note that full information on the approval of the study protocol must also be provided in the manuscript.

Field-specific reporting

Please select the one below that is the best fit for your research. If you are not sure, read the appropriate sections before making your selection.

☒ Life sciences ☐ Behavioural & social sciences ☐ Ecological, evolutionary & environmental sciences

For a reference copy of the document with all sections, see nature.com/documents/nr-reporting-summary-flat.pdf

Life sciences study design

All studies must disclose on these points even when the disclosure is negative.

Sample size	No sample calculations were performed. Sample sizes were dependent on breeding colony numbers. Experiment included 5 or more independent samples for most studies.
Data exclusions	We excluded outliers in the Iba-1+ flat mounts. For the analysis of Iba-1+ cells in flat mounts we provide the P-value both including all values (black asterisks) and also excluding outliers (blue asterisks). We used the interquartile range (IQR) to determine outliers, which were defined as those outside of a range going from Q1–1.5*IQR to Q3+1.5*IQR, where Q1 and Q3 are the first and third quartiles, respectively.
Replication	Key experiments were replicated in the founder group and then in an expanded colony group.
Randomization	Randomization was not required for our experiments. There were no therapeutic interventions. All animals for a given age group for each genetic group were analyzed. However, masking during testing and analysis was performed (see below).
Blinding	Measurements were done by investigators masked to the genotype of the mice.

Reporting for specific materials, systems and methods

We require information from authors about some types of materials, experimental systems and methods used in many studies. Here, indicate whether each material, system or method listed is relevant to your study. If you are not sure if a list item applies to your research, read the appropriate section before selecting a response.

Materials & experimental systems

n/a	Involved in the study	
☐	☒	Antibodies
☒	☐	Eukaryotic cell lines
☒	☐	Palaeontology and archaeology
☐	☒	Animals and other organisms
☒	☐	Clinical data
☒	☐	Dual use research of concern

Methods

n/a	Involved in the study	
☒	☐	ChIP-seq
☒	☐	Flow cytometry
☒	☐	MRI-based neuroimaging

Antibodies

Antibodies used

1. IBA-1 Polyclonal, Wako Chemicals USA, Cat. # 019-19741
2. CD16/CD32 Polyclonal, BD pharmaceutical, Cat. # 553142
3. Lipe/HSL Polyclonal, Cell Signaling Technology, Cat. # 4107S
4. Cone Arrestin, Sigma-Aldrich, Cat. # Ab15282
5. TMEM119, Novus Biologicals, LLC, Cat. # NBP3-13355
6. CCR2, Novus Biologicals, LLC, Cat. # NBP2-35334
7. F4/80, ThermoFisher Scientific, Cat. # 14-4801-81

Validation

The Iba-1, CD16, TMEM119, CCR2, F4/80 and cone arrestin antibodies have been validated by the companies, and also extensively used in publications looking at microglia in the CNS and at the retina by many groups, including ours (PMID 25588310 and 26030099). Lipe antibody was validated by Cell Signaling Technologies (https://www.cellsignal.com/products/primary-antibodies/hsl-antibody/4107). Also, our manuscript includes Western Blot data that corroborates that the antibody stains a protein of the correct size in WT mice, but not in Lipe KO mice.

Animals and other research organisms

Policy information about studies involving animals; ARRIVE guidelines recommended for reporting animal research, and Sex and Gender in Research

Laboratory animals

C57Bl/6J mice from Jackson Laboratories, CRISPR-Cas9-generated Lipe KO mice developed on a B6J background and littermate controls

Wild animals

NA

Reporting on sex

Sex was considered as a biological variable. Before combining data from male and female mice we confirmed that our basic findings (OCT changes and retinal fundus spot changes) were not different when comparing males and females. As expected, weight was different between males and females. We also observed differences in serum lipid profiles in males vs. females, and these were analyzed and reported.

Field-collected samples

NA

Ethics oversight

All studies were reviewed and approved by the Institutional Animal Care and Use Committee at UT Southwestern Medical Center.

Note that full information on the approval of the study protocol must also be provided in the manuscript.
